# Modelling the burden of disease for cattle–A case of ticks and tick-borne diseases in cattle in a rural set-up in South Africa

**DOI:** 10.1371/journal.pone.0293005

**Published:** 2023-10-20

**Authors:** Omran Salih, Simbarashe Chitanga, Keshlan Govinder, Samson Mukaratirwa

**Affiliations:** 1 School of Mathematics, Statistics and Computer Science, University of KwaZulu Natal, Durban, South Africa; 2 Department of Mathematical Sciences, Faculty of Education, The University of Alzaiem Alazhari, Khartoum, Sudan; 3 Institute of Systems Science, Durban University of Technology, Durban, South Africa; 4 School of Life Sciences, University of KwaZulu-Natal, Durban, South Africa; 5 Department of Biomedical Sciences, School of Health Sciences, The University of Zambia, Lusaka, Zambia; 6 Department of Preclinical Studies, School of Veterinary Medicine, University of Namibia, Windhoek, Namibia; 7 One Health Center for Zoonoses and Tropical Veterinary Medicine, School of Veterinary Medicine, Ross University, Basseterre, Saint Kitts and Nevis; Bangladesh Agricultural University, BANGLADESH

## Abstract

**Background:**

The study aimed to estimate the burden of ticks and tick-borne diseases (TBDs) among rural cattle-keeping households of the Eastern Cape province of South Africa using Productivity Adjusted Life Years (PALYs). We modified Disability Adjusted Life Year (DALY) equations for humans to PALYs to estimate the societal burden of tick-borne animal diseases. Whilst the World Health Organization has indicated the adaptability of DALYs to assess burden of animal diseases, nothing has been done in this regard. This could be due to several reasons including that the assessment of animal disease burden is often less of a priority compared to human diseases, particularly in low- and middle-income countries where resources may be limited. As a result, less funding and attention may be given to developing and implementing PALYs for animal diseases. Furthermore, technical and conceptual challenges may be associated with applying DALYs equations to animal diseases, such as determining appropriate measures of productivity loss for different types and categories of animals and diseases. This motivated our study, which is focused on modelling the burden of ticks and TBDs in cattle (cows, oxen and bulls) reared in resource-poor settings.

**Method:**

We formulated a PALYs approach for cattle populations by adapting the DALYs approach to assess the burden of ticks and TBDs for cattle populations in 20 villages in the Eastern Cape province of South Africa. PALYs is a measurement used to assess the burden of disease in cattle populations, quantifying the years of life lost due to premature mortality and disability. It encompasses years of life lost due to premature mortality (YLL) and years lost due to disability (YLD) caused by health conditions. PALYs provide a comprehensive perspective on the effective number of years lost due to disability and premature death in cattle populations. The PALYs model involves several parameters that are examined to understand their impact on the model’s behavior. To illustrate this, we used a structured questionnaire to collect data on parameters that feed into PALY equations. We coded and entered data from questionnaires directly into Statistical Package of Social Sciences (IBM SPSS Version 20) and entered the estimated values of PALY parameters to calculate PALYs equations, which were to estimate the societal disease burden of ticks and TBDs in cattle. PALYs calculations were done in three categories; PALYs without discounting and age weighting, PALYs with only discounting, and PALYs with discounting and age weighting in a practical example to study how these parameters influence the outcomes of the PALYs model.

**Results:**

Our results revealed that the years of productivity lost by a cow, bull, and ox that suffered from ticks and TBDs could be estimated at various disability weights. Approximately 26%, 23% and 35% of the productivity years of a cow, ox and bull, respectively, reared by resource-poor livestock owners are lost due to the burden of ticks and TBDs in the Eastern Cape province of South Africa. However, introducing tick control measures reduces the loss to approximately 3%, 2% and 3% of their lifespan productivity, an indication that tick control will save approximately 23%, 21% and 32% of years of the productive life of cows, oxen and bulls, respectively. Therefore, it is evident that using ticks and TBD prevention measures at an early age of cattle will improve cattle productivity and hence the socioeconomic welfare of resource-poor rural farming communities in the Eastern Cape province of South Africa.

**Conclusion:**

The findings generated from the PALYs approach are helpful in projections for the future burden of any livestock disease. They may be used as a basis in policy formulation and decision-making by various stakeholders, and hence a priority in animal health economics. We recommend that a classification of livestock diseases of national economic importance should consider both the societal burden (non-monetary) and economic impact instead of the common practice of only considering the economic (monetary) impact. Adding a societal burden measure to existing economic measures provides a holistic understanding of the impact of a disease on society especially in resource-limited settings where the livestock value goes beyond monetary value.

## Introduction

The World Health Organization quantifies burden of disease by using summary measures of population health (SMPH) [1] which consider both non-fatal health and mortality outcomes. This is represented as a single number and then used as a yardstick to determine the health of a particular population. The task of developing SMPH has a long history, refined over time through the efforts of researchers and organizations. It provides a comprehensive and easy-to-understand measure of overall population health, which can assist policymakers and decision-makers in identifying intervention areas and tracking progress in improving population health over time [2–6]. SMPH has many applications which include the active life expectancy [7] undertaken in the United States of America, quality adjusted life years by the global burden of disease [8], the disability free life expectancy [9, 10] and the Disability Adjusted Life Years (DALYs). DALYs are widely used by global disease burden studies [11–13] and national burden of disease studies [12, 14, 15]. Whilst livestock, especially cattle, play an important role in human health and well-being as source of income, meat, milk, draught power, employment and as a measurement of wealth/social status [16], to date, no attempts have been made to adapt these measures in quantifying disease burden in livestock.

In sub-Saharan African countries, including South Africa, apart from cattle providing meat, milk and hide production [17], they also have social significance/value which include status symbolism and playing a role during religious/cultural-practices [18]. For rural livestock owners, cattle production is also important for crop production through provision of draught power and manure for their crops. Thus, cattle production in a rural set-up is critical and contributes to multiple livelihoods and offers alternative pathways out of poverty.

Ticks and TBDs are among the group of pests and diseases which have serious impact on cattle health and production in the tropical regions, with their impact being influenced by the type of production system as well as the agroecological settings. In the rural Eastern Cape province of South Africa which practices extensive farming, cattle farming is severely hampered by ticks and TBDs; with anaplasmosis (gall-sickness), babesiosis (red water) and ehrlichiosis (heart water) being considered among the most severe TBDs [19].

The social and economic burden of a disease in livestock generally refers to the effect a disease has on society, measured in terms of mortality, morbidity, financial cost, or other indicators [20]. These variables enable assessment of the impact of a disease and the easiest metric used to assess the impact is the financial cost. However, in a resource poor community, the value of livestock is not only considered at economic/monetary terms, as livestock also serve several social and cultural responsibilities which are of great value to the owner/community. Therefore, when cattle losses occur due to disease, the impact cuts across all societal levels and hence its direct costs need to be estimated using market prices. The indirect costs associated with cattle loss are more difficult to estimate even though impact could be more relevant for rural poor-resourced livestock owners than the direct financial costs [21].

To estimate the economic and social values of the burden of ticks and TBDs, certain methods ought to be sought to combine the adverse effects of the diseases on the community and reduced productivity in cattle in conjunction with the low levels of human well-being and productivity. The direct costs are related to the damage produced by the ticks while feeding on their hosts. In contrast, the indirect losses are related to the infectious agents transmitted by the ticks and the costs associated with the treatment and control.

To date, there are no standard mathematical methods to measure and assess the impact of ticks and TBDs on livestock, affecting the health and productivity of cattle. The most common methods are statistical, and the acceptable procedure is data collection about a specific disease and then followed by modeling using preferred statistical techniques [16, 22]. For example, using such techniques as least squares regressions requires large data sets to obtain realistic conclusions. Moreover, the results obtained via regression analysis are heavily dependent on the respective formulated form if the error term is inadequately interpreted. This gives varying calculations that are dependent on how the regression is initially set up. When using corrected least squares regressions, the results are affected by outliers, since the “best” performer along any dimension serves as the anchor for the estimate.

The challenges with statistical methods, cited above, motivated us to develop mathematical methods for this study. We modified the DALYs model [23–25] to measure PALYs and then assessed the burden of disease for livestock. This measure of cattle population health combines mortality and morbidity into one unit to measure the impact of a disease on communities. The reason for using the DALYs approach to formulate a PALYs approach for cattle populations is because the DALYs model has been widely used in human health assessment to successfully give realistic results. Thus, we expect our new model to give realistic results compared to statistical methods.

## PALYs for cattle population

Our aim in this section was to reformulate the DALYs model for human population into a productivity adjusted life years (PALYs) model for cattle to be used to assess the burden of ticks and TBDs on cattle production. We used a structured questionnaire to collect the required information to feed into the PALY equations. The study was approved by the Biomedical Research Ethics Committee of the University of KwaZulu-Natal (BE109/14). Written consent to participate in the study was sought from the farmers before participation in the study. For individuals who could neither read nor write, the consent was read out to them by an enumerator in the local language, and they appended their thumb print in lieu of the signature.

We coded and entered data from questionnaires directly into Statistical Package of Social Sciences (IBM SPSS Version 20) and entered the estimated values of PALY parameters to calculate PALYs equations The values were used to estimate the societal disease burden of ticks and TBDs in cattle. PALYs calculations were done in three categories; PALYs without discounting and age weighting, PALYs with only discounting, and PALYs with discounting and age weighting in a practical example to study how these parameters influence the outcomes of the PALYs model.

### PALYs concept for cattle

In constructing a PALYs model for cattle, several aspects were readjusted or reconfigured from the standard DALYs model. In building the PALYs model, the following four choices were redefined:

1. Standard lifespan,
2. Disability weight,
3. Discounting,
4. Age weighting.

Considering that there is limited information available on these parameters, and the context specificity of any such information, we collected information specific to the Eastern Cape province of South Africa using a carefully designed questionnaire (See S1 File).

### Data collection

A questionnaire was designed in such a way that participants directly involved in looking after the cattle were interviewed in their local language by a trained interpreter. The obtained information was analyzed to determine the importance of social, economic values and impact of ticks and TBDs. This information was also used to calculate a standard lifespan table for cattle and deduce a definition of disability weight for cattle and the age weighting function. A copy of the collected data is attached in S2 File.

The social and economic values of cattle and economic impact of diseases on cattle production in Eastern Cape Province of South Africa was examined using a probabilistic sampling method [26]. A simple random sampling of 20 villages from Eastern Cape province was selected, and 18 livestock owners from each village were interviewed. The communal farming areas in the Eastern Cape province consist of villages with a residential area, cropping area, and communal grazing area. Each household has a residential area, garden, and cropping field and shared grazing land for their livestock. Livestock, which includes cattle, sheep, goats, pigs, horses, and donkeys, are considered an investment, and sold when money is needed [27]. Furthermore, rural dwellers keep livestock for cultural and traditional reasons, as they symbolize wealth, prestige, and social status [28].

The sample size of the livestock owners was calculated using an online sample size calculator [29] based on the following assumption: population size of 75,000 livestock owners, 95% confidence level and 5% for confidence interval. The Department of Agriculture in the Eastern Cape province facilitated the interactions with the identified livestock owners. The sample size formula used can be found in S3 File.

### Lifespan

Lifespan for cattle was defined as the “expected number of years of life remaining at a given age”. We used an approach that has been used in calculating lifespan for human populations in many countries [30], by making use of the *l*_*x*_ table [25, 31, 32]. In calculating lifespan *l*_*x*_ table for cattle, the following definitions were used

- *x* is age group (*x* = 1,2,⋯,*k*).
- *n*_*x*_ is the number of cattle at age *x* which survived until the age *x*.
- *d*_*x*_ is the death rate, this refers to the number of cattle that died at age *x*. The death rate *d*_*x*_ is evaluated as follows
$$d_{x}=n_{x}-n_{x+1};\;\sum_{i=0}^{k}d_{i}=n_{0}$$
- *l*_*x*_ is the probability of cattle population that survives at age *x* (survivorship). This is given by
$$l_{x}=\frac{n_{x}}{n_{0}}$$
- *q*_*x*_ is the probability of dying at age *x* (e.g between ages *x* and *x*+1). This is given by
$$q_{x}=\frac{d_{x}}{n_{x}};\;q_{k-1}=1.$$
- *L*_*x*_ is midpoint of probability of the cattle population that survives at age *x* (midpoint survivorship). That is,
$$L_{x}=\frac{\left(l_{x}-l_{x+1}\right)}{2}.$$
  Note that the sum of $\sum_{i=0}^{k}L_{x}$ gives the total probability of age groups lived by the entire study population.
- *T*_*x*_ is the total probability of age groups left to be survived by population who survive at age *x*,
$$T_{x}=T_{x-1}-L_{x-1};\;T_{0}=\sum_{i=0}^{k}L_{x}.$$
- *e*_*x*_, defined as the expectancy at age *x* is given by the following equation
$$e_{x}=\frac{T_{x}}{l_{x}};\;e_{k-1}=\frac{1}{2}.$$

The calculation of the standard lifespan using the *l*_*x*_ table requires knowing the number of dead animals due to TBDs or old age and at what age. We obtained this information by inquiring as to the total animals lost in a year and then the age at death.

### Disability weight

Disability is defined as some form of inability to perform daily tasks or rituals in a way that is usual for cattle kept by a designated individual or community. Disability weight is a weight function that reflects the severity of a disease of cattle between 0 (perfect health) and 1 (equivalent to death). Disability health outcomes of cattle diseases differ depending on the cause, nature, and the impact on the individual cattle.

We derived the definition of the disability weight for cattle from their social and economic values. This is because the sole purpose for keeping cattle is the benefits that humans derive from them. Therefore, we have formulated a definition of disability weight for cattle so that each disease can be assigned a specific number between 0 and 1. These weights were then grouped into four levels as illustrated in the third column of S1 Table This table also gives the general classification that we adopted as regards to how the disease affects the productivity derived from cattle.

The parameters we considered to evaluate the impact or severity of the disease on cattle are beef production, milk production, draught power, social status, dowry payment and cultural ceremonies. We asked livestock owners why they kept animals and what was the impact of ticks and TBDs on cattle with respect to these six parameters. These responses, taken together with other published data [16], enabled us to classify the impact of the diseases. The first question allowed us to determine the purpose of keeping the cattle with reference to our parameters and the second question revealed the impact of ticks and TBDs in each parameter.

According to this data, we were able to weigh the severity of a set of indicators disabling conditions, that causes an unhealthy status. We grouped these weights into four levels according to outcome of the impact of the disease. In level 1, we have perfect health. Level 2 has weights between 0.01–0.33, level 3 has weights between 0.34–0.66 and level 4 has weights between 0.67–0.99. If there is change of any of the parameters (beef production, milk production, draught power, social status, dowry payment, and cultural ceremonies), then the disability weight should be changed. For example, assume that a dairy cow becomes sick due to a particular disease and the disease condition changes its milk production from 6 liters per day to 3 liters per day, then the disability weight for this condition should be between 0.34 and 0.66.

With regards to dowry, the body condition of the animal was the main consideration, with healthy animals being the most ideal. A diseased animal with poor body condition was the least acceptable for dowry thus we placed it into category 4 for the disability weight scale. Since optimal weight for beef productivity required a weight of 500–600 kg for oxen [33], this was classified as level 1 and the disability weight was 0. Based on this, a loss of 100 kg resulted in the animal dropping a level on the disability weight scale to level 2. Subsequent lowering of levels of disability weight were also by 100 kg weight increments.

### Discounting

**Fig 1:**
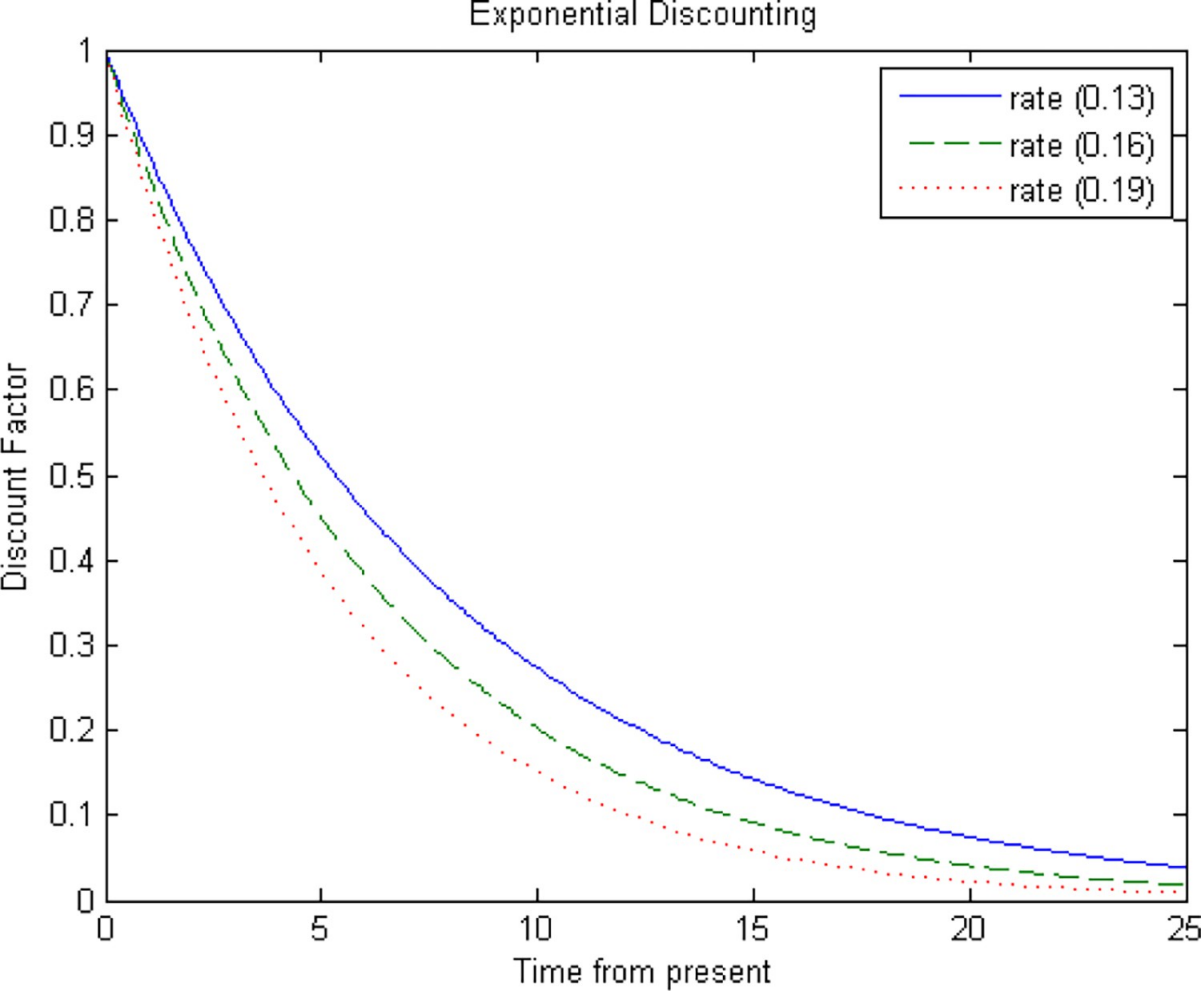
Discounting effects on PALYs: A comparison of different rates.

In this study, we used the same discount function (exponential decay) used in the DALYs model for human population. However, we changed the discount rate to be able to obtain the same effect of discounting in the number of years of life lost at a different time in the future.

(Fig 1) shows the effect of discounting in the value of a year of life lost at different times in the future for cattle. For the purpose of calculation, the PALYs formulae for cattle used a continuous function given by the following equation:

$$G(x)=e^{rx},$$ (1)

where *G*(*x*) is a continuous discounting function at any age *x* and *r*>0 is the discount rate.

### Age-weighting

**Fig 2:**
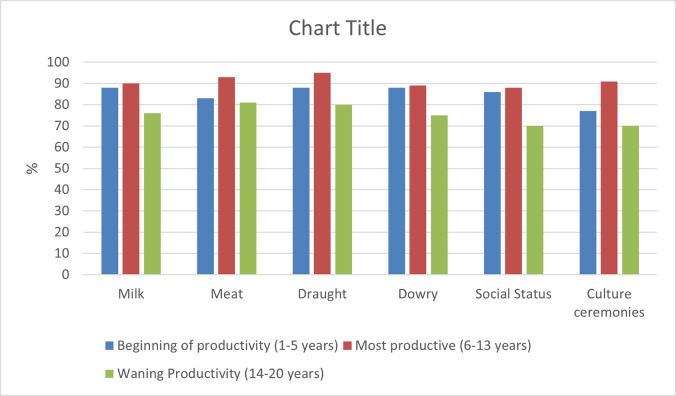
Productivity age for cattle amongst rural livestock owners in the Eastern Cape province, South Africa (2013).

**Fig 3:**
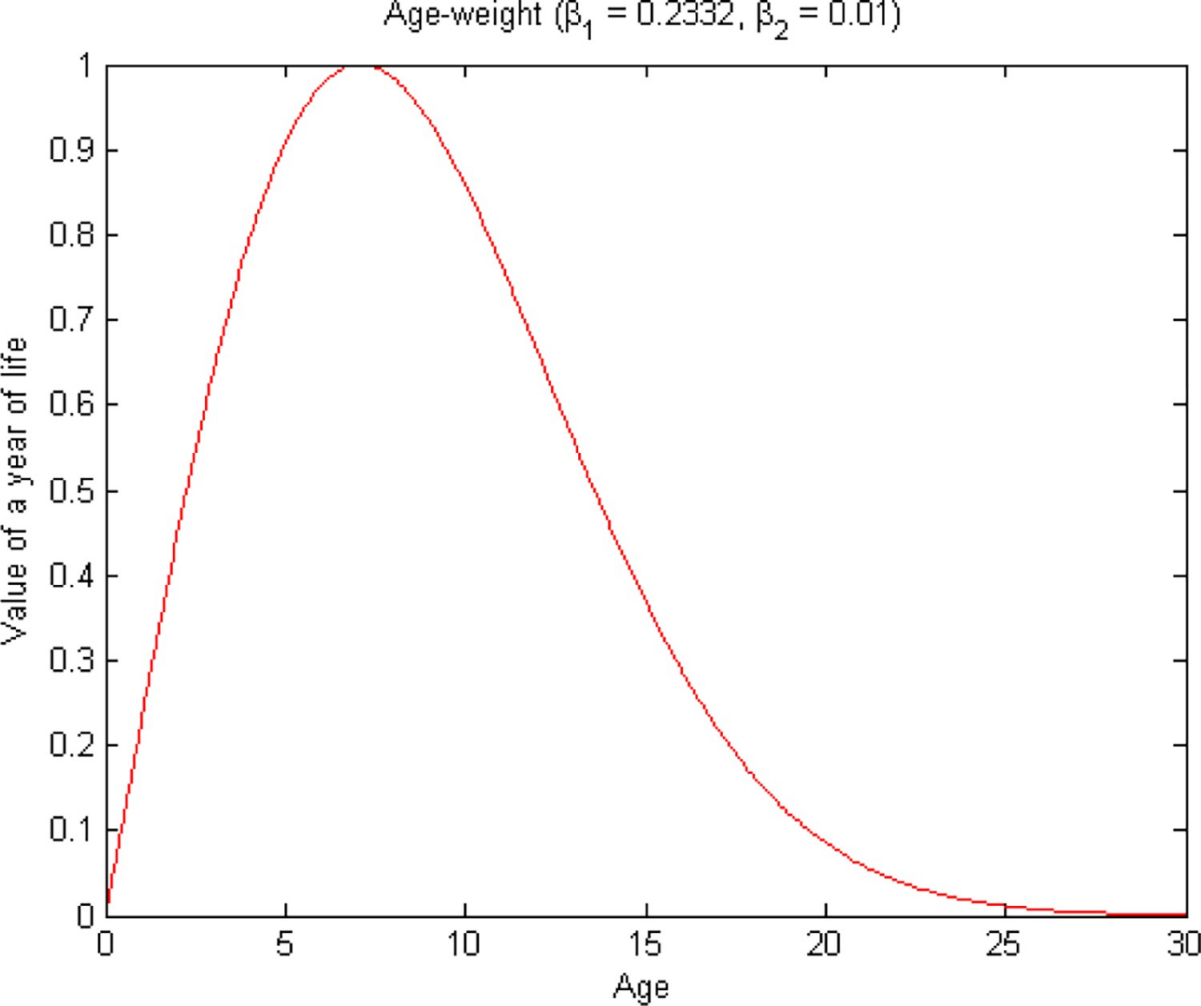
Values of productivity for cattle in different age groups amongst rural livestock owners in Eastern Cape province, South Africa (2013).

Here we wished to determine the age at which cattle start and stop being of value or productive. We asked livestock owners the age at which animals become productive in terms of meat, milk, draught power, social status, dowry, and cultural ceremonies. (Fig 2) represents their responses as a percentage of age productivity. This data shows the age of productivity of years of life for different types of products. For example, the percentage of cattle which start milk production between ages 1 to 5 is 87% and that of meat is 91%. According to our data it is clear that as a particular member of a cattle population grows towards the productive age, its life becomes more valuable (the age weight increase) until it reaches its maximum at the expected age of maximum productivity. Then as it gets older, its life gradually loses value (Fig 3).

Age weighting means that the life years of cattle are counted differently because cattle are more productive at a particular age than others. In this study, age-weight is based on the questionnaire which has indicated that there is a broad variation in economic and social values of cattle at different ages. The impact of lost years of a healthy life varies significantly with cattle ages; for instance, lost years of healthy life during the productivity ages has a greater negative impact compared with lost years of healthy life during the very early age or late age. According to our data, we valued years of healthy life lived during productive ages over early and late ages. We consider this choice reasonable, and it is made based on economic and social values. Using (Fig 2), we represented an age weighting curve as indicated in (Fig 3). The relative value of a life year is below 0.8 for age under 5 as well as for cattle older than 13 years of age. This implies that, in the calculations, the value of a life year lost is weighted the same in both regions. The preference of productive ages shown by the function plotted in (Fig 3) can be expressed mathematically as

$$R(x)=\beta_{1}xe^{-\beta_{2}x^{2}},$$ (2)

where *x* is the age of the cattle, while *β*1 and *β*2 are parameters of the age weighting function. Fixing the maximum of *R*(*x*) = 1 (because we choose to take our age-weighting scale from zero to one) yields

$$\beta_{1}=\sqrt{2\beta_{2}}e^{\frac{1}{2}},\;\beta_{2}>0.$$

In the above equation, *β*_1_ determines the importance of age-weights and is chosen arbitrarily; *β*_2_ is an adjustment constant, chosen so that the introduction of age-weights does not alter the total number of years of life lost. The value of *β*_1_ = 0.2332 and *β*_2_ = 0.01 used in our PALY calculation.

### Calculating PALYs for cattle

PALYs for a disease or health condition are calculated as the sum of the years of life lost due to premature mortality (YLL) in the cattle population and the equivalent ‘healthy’ years lost due to disability (YLD) for incident cases of the health condition:

PALYsforcattle=YLLforcattle+YLDforcattle. (3)

### Basic formulas for YLD and YLL

The basic formula for YLD (without age weighting and discounting) is basically the product of the number of disability cases, the average duration of the disease and the disability weight. This translates to

$$YLD\;for\;cattle=N_{i}\times D_{w}\times I,$$ (4)

where *N*_*i*_ is the number of incident cases, *D*_*w*_ is the disability weight, and *I* is the average duration of disability.

The basic formula for YLL (without age weighting and discounting) is defined as the product of the number of deaths and the standard lifespan at the age of mortality. This becomes

$$YLL\;for\;cattle=N_{d}\times L,$$ (5)

where *N*_*d*_ is the number of deaths and *L* is the standard lifespan at age of death. Note that both formulae do not change whether we refer to humans or animals. However, the parameters values are different.

### YLD and YLL with discounting

Whilst there are different methods for calculating YLD and YLL, we used the formula that considers the discount function. We obtained the formula for YLD by multiplying Eqs (4) and (1).

$$YLD\;for\;cattle=N_{i}\times D_{w}\times I\times G(x),$$ (6)

where *G*(*x*) is the discounting function. The total discounting in the continuous-time case is given by

$$TD=\int_{a_{i}}^{a_{i}+I}e^{-r(x-a_{i})}dx$$ (7)

where *a*_*i*_ is the age of onset. By substituting Eq (7) into Eq (6), the total YLD in the continuous-time case becomes

$$YLD\;for\;\;cattle=\int_{a_{i}}^{a_{i}+I}N_{i}D_{w}e^{-r(x-a_{i})}dx$$ (8)

which evaluates to

$$YLD\;for\;cattle=\frac{N_{i}D_{w}[1-e^{-rI}]}{r},$$ (9)

where *r* is the discount rate.

Similarly, to find a formula for the YLL, we simply modified Eq (9) by setting the disability weight to 1 (*D*_*w*_ = 1) and replacing the average duration of disease (*I*) by the standard lifespan at the age of death (*L*). The YLL formula is then

$$YLL\;for\;cattle=\frac{N_{d}[1-e^{-rL}]}{r},$$ (10)

where *N*_*d*_ is the number of deaths.

### YLD and YLL with discounting and age-weighting

The YLD value of any disability weight (*D*_*w*_) with discounting function, age-weighting function and number of disease cases (*N*_*i*_), is given as:

$$YLD\;for\;cattle=N_{i}\times D_{w}\times G(x)\times R(x)$$ (11)

where *G*(*x*) is the discounting function and *R*(*x*) is the age-weighting function.

To calculate the YLD that accounts for the duration of life lost due to disability, 2duration from the age of onset, we simply integrated the disability weight times the age weight and discount function over the expected period of the disability. By substituting Eq (7) and (2) into Eq (11), we yielded

$$YLD\;for\;cattle=\int_{a_{i}}^{a_{i}+I}N_{i}D_{w}\beta_{1}xe^{-\beta_{2}x^{2}}e^{-r(x-a_{i})}dx$$ (12)

where *I* is average duration of disability and *a*_*i*_ is the age of onset. Eq (12) can be evaluated as

$$YLD\;for\;cattle=N_{i}D_{w}\beta_{1}e^{a_{i}r}\left[\sqrt{\pi }re^{\frac{r^{2}}{4\beta_{2}}}(-\frac{\mathrm{erf}(\frac{2\beta_{2}(a_{i}+I)+r}{2\sqrt{\beta_{2}}})+\mathrm{erf}\left(\frac{2\beta_{2}a_{i}+r}{2\sqrt{\beta_{2}}}\right)}{4\sqrt{\beta_{2}^{3}}})+(\frac{-e^{-(a_{i}+I)(\beta_{2}(a_{i}+I)+r)}+e^{-a_{i}(\beta_{2}a_{i}+r)}}{2\beta_{2}})\right],$$ (13)

where *N*_*i*_ is the number of incident cases, *D*_*w*_ is the disability weight, *I* is the average duration of disability, *r* is the discount rate, *a*_*i*_ is the age of onset and erf is the error function. Typical values of *β*_1_ and *β*_2_ are 0.2332 and 0.01 respectively.

Similarly, by replacing the duration of disease (*I*) with the standard lifespan (*L*), the age onset (*a*_*i*_) with the age of death (*a*_*d*_), and setting the disability weight to one in Eq (13), we obtain the YLL formula as

$$YLL\;for\;cattle=N_{d}\beta_{1}e^{a_{d}r}\left[\sqrt{\pi }re^{\frac{r^{2}}{4\beta_{2}}}(-\frac{\mathrm{erf}(\frac{2\beta_{2}(a_{d}+L)+r}{2\sqrt{\beta_{2}}})+\mathrm{erf}\left(\frac{2\beta_{2}a_{d}+r}{2\sqrt{\beta_{2}}}\right)}{4\sqrt{\beta_{2}^{3}}})+(\frac{-e^{-(a_{d}+L)(\beta_{2}(a_{d}+L)+r)}+e^{-a_{i}(\beta_{2}a_{d}+r)}}{2\beta_{2}})\right],$$ (14)

where *N*_*d*_ is the number of deaths, *a*_*d*_ is the age of death, *L* is the standard lifespan at age of death.

In this guide, we presented the basic procedure for calculating PALYs for cattle using a suitable example (and provided an illustrative example demonstrating the process with and without considering discounting rate and age weighting. We also summarized the key findings of our analysis, presenting the PALY values obtained under different scenarios considered (See S4 File).

In this section, we calculated the standard lifespan for cattle and defined the disability weight for cattle diseases. In addition, we shed light on the age weighting of cattle. Furthermore, we built a new model for cattle populations to assess the burden of diseases. We demonstrated the applicability of our model with a worked-out example. The example clearly illustrated the value in incorporating age weighting and discounting into a realistic PALYs model.

## Results

To assess the impact of Ticks and TBDs, we applied the PALYs model for these animals considering discounting and age weighting. We also shown how many PALYs can be averted when the ticks are controlled.

**Fig 4:**
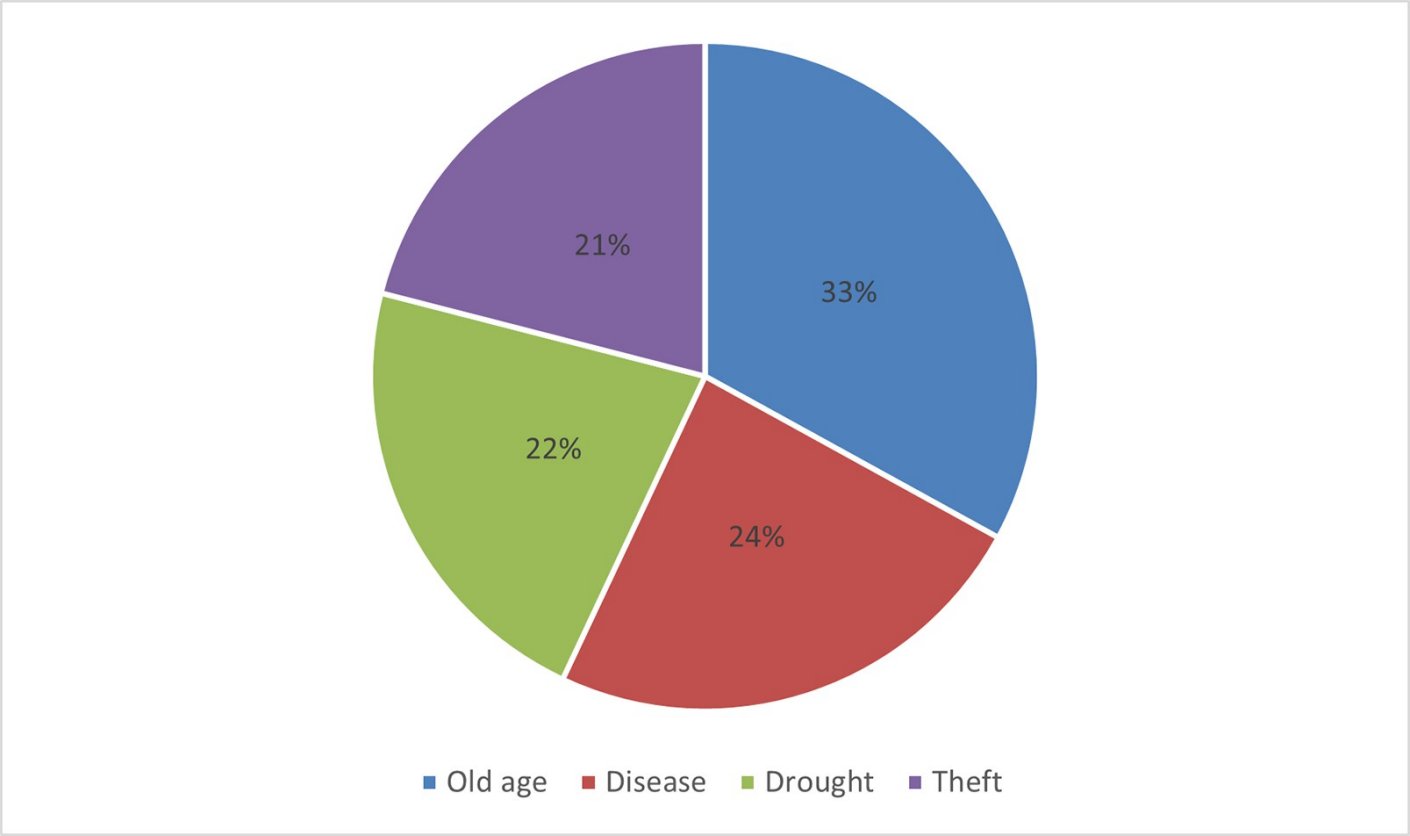
Most common causes of cattle mortality amongst rural livestock owners in Eastern Cape province, South Africa (2013).

From our questionnaire survey, livestock deaths were mostly ascribed to old age, disease, drought and theft, with relative frequencies of 33%, 24%, 22% and 21%, respectively (Fig 4).

In response to the question on numbers of animals that had died and at which age death occurred, we found that a total of 116 cows, 124 oxen and 86 bulls had died, however, the livestock owners could not respond to the ages at which deaths had occurred. Hence, we theoretically estimated the number of animals that died at different ages using a survival distribution function as previously described by Chen et al. [5] (Table 1). The data obtained from the interviews was then used to determine the PALYs, based on the severity of the disease.

**Table 1:** The number of dead animals in each age-group amongst rural livestock owners in Eastern Cape province, South Africa (2013).

Age Group	Cows		Oxen		Bulls	
	Percent	Numbers	Percent	Numbers	Percent	Numbers
0–4	5%	6	5%	6	5%	4
5–9	15%	17	15%	19	15%	13
10–14	20%	23	20%	25	19%	17
15–19	25%	29	25%	31	25%	22
20+	35%	41	35%	43	36%	31
Total		116		124		86

S2 Table illustrated the steps taken in calculating the standard lifespan of the cattle population (cows) from mortality data. In S3 Table, we only quote the standard lifespan for bulls and oxen. For example, if a bull is 5 years old, then its lifespan at this age is 9.39 years. It is interesting to note that the standard lifespan for cattle using the *l*_*x*_ table is greater than using statistical estimation based on the livestock owners’ responses.

The results for PALYs were derived using the YLL and YLD formulas as outlined in Eqs (13) and (14) of our methodology. Once we calculated the YLL and YLD values, we obtained the PALYs by adding them together. Detailed conceptual and computational analysis on how the YLD and YLL for individual conditions were estimated is presented previously.

### PALYs for cows: No controls

Sample data in Table 1 was used to estimate PALYs for the cow population in this region at disability levels 2, 3 and 4.

In our calculations we assumed *D*_*w*_ and *I* to be constants for each animal type at a given level of disability. This is because, for *D*_*w*_, we assume that all the animals are affected by the same disease, and in the case of *I*, that it is not easy for the farmer to estimate the average time and animal spends with the disease before it dies.

In Table 2, we calculated YLD, YLL and PALYs with *D*_*w*_ = 0.33. At this level, the YLD is on average 0.32 years (approximately 3 months and 3 weeks). The YLL is approximately 2.88 years (approximately 35 months). The number of PALYs lost for the 116 cows when in level 2 is 370.20 years. Hence, on average, a farmer loses 3.18 years (approximately 38 months and 3 weeks) of productivity per cow due to fatality of the disease.

**Table 2:** PALYs for cows with *D*_*w*_ = 0.33.

Age group	*N*_*i*_	*a*_*i*_	*I*	*D*_*w*_	YLD	*N*_*d*_	*a*_*d*_	*L*	YLL	PALYs
0–4	6	2	1	0.33	4.18	6	3	11.51	71.14	75.32
5–9	17	7	1	0.33	11.77	17	8	9.29	141.41	153.18
10–15	23	12	1	0.33	10.88	23	13	7.01	92.94	103.82
16–20	29	18	1	0.33	7.75	29	19	3.50	20.51	28.26
20 +	41	20	1	0.33	2.06	41	21	1.50	7.56	9.62
Total	116				36.64	116			333.56	370.20

Table 3 shows the numbers of YLD, YLL and PALYs lost for the 116 cows when *D*_*w*_ = 0.52. At this level, the YLD is on average 0.45 years (approximately 5 months and 2 weeks). The YLL is approximately 2.88 years (approximately 35 months). The number of PALYs lost for 116 cows is 385.54 years, therefore the average PALYs lost per cow is 3.33 years (approximately 40 months and 2 weeks).

**Table 3:** PALYs for cows with *D*_*w*_ = 0.52.

Age group	*N*_*i*_	*a*_*i*_	*I*	*D*_*w*_	YLD	*N*_*d*_	*a*_*d*_	*L*	YLL	PALYs
0–4	6	2	1	0.52	6.08	6	3	11.51	71.14	77.22
5–9	17	7	1	0.52	17.48	17	8	9.29	141.41	158.89
10–14	23	12	1	0.52	16.16	23	13	7.01	92.94	109.10
15–19	29	18	1	0.52	7.06	29	19	3.50	20.51	27.57
20+	41	20	1	0.52	5.20	41	21	1.50	7.56	12.76
Total	116				51.98	116			333.56	385.54

Table 4 shows the calculation of PALYs lost for 116 cows when considering disability weight from level 4 (*D*_*w*_ = 0.84). At this level, the YLD is on average 0.72 years (approximately 8 months and 3 weeks). The YLL is approximately 2.88 years (approximately 35 months). The number of PALYs lost for 116 cows is 417.54 years, therefore the average PALYs lost per cow is 3.60 years (approximately 43 months 3 weeks). This is equivalent to 23% of a cow’s lifespan at birth.

**Table 4:** PALYs for cows with *D*_*w*_ = 0.84.

Age group	*N*_*i*_	*a*_*i*_	*I*	*D*_*w*_	YLD	*N*_*d*_	*a*_*d*_	*L*	YLL	PALYs
0–4	6	2	1	0.84	9.83	6	3	11.51	71.14	80.97
5–9	17	7	1	0.84	28.24	17	8	9.29	141.41	169.65
10–15	23	12	1	0.84	26.10	23	13	7.01	92.94	119.04
16–20	29	18	1	0.84	11.40	29	19	3.50	20.51	31.91
20 +	41	20	1	0.84	8.41	41	21	1.50	7.56	15.97
Total	116				83.98	116			333.56	417.54

### PALYs for cows: Tick control

The previous calculations of PALYs were done without considering intervention methods for tick control. We therefore sought to determine the PALYs gained when tick control methods are adopted. In what follows we assume that the cows received treatment for their TBDs at the age of onset (*a*_*i*_), and as a result did not die at the age of expected death (*a*_*d*_) but lived for their expected life span at the age *a*_*i*_ (in the treated state). With these assumptions, the disability weight for the treated TBDs is 0.10 (Tick control measures for TBDs can significantly reduce disability weight, lowering it to 0.01. Such measures enable cattle to resume their daily activities without hindrance.) (S1 Table). Thereafter, the YLD with treatment was calculated by changing the disability weight for the treated form of the disease from 0.33 or 0.52 or 0.84 (without treatment) to 0.10 (with treatment) (Table 5). In this case the cows (and the other animals in general) would have lived for their expected life at age of onset.

**Table 5:** PALYs for 116 cows with tick control in Eastern Cape province, 2013.

Age group	*N*_*i*_	*a*_*i*_	*I*	*D*_*w*_	YLD	PALYs
0–4	6	2	11.94	0.10	6.17	6.17
5–9	17	7	9.70	0.10	13.96	13.96
10–15	23	12	7.38	0.10	10.51	10.51
16–20	29	18	4.80	0.10	4.06	4.06
20 +	41	20	2.90	0.10	2.83	2.83
Total	116				37.53	37.53

Table 6 shows the PALYs calculations with tick control. The PALYs lost per cow during the treatment is 0.32 years (approximately 3 months and 26 days). This clearly shows that when cows are given tick control treatment, their disability weight is reduced to 0.10, indicating that the treatment significantly improves their overall health and well-being. In Table 6, we present the PALYs with and without treatment for the individual cattle (cow). In the last column we highlight the PALYs averted as a result of tick control.

**Table 6:** PALYs averted per cow in Eastern Cape province 2013.

*D*_*w*_	PALYs (treatment)	PALYs (no treatment)	PALYs averted
0.35	3.18 years	0.32 years	2.86 years
0.52	3.31 years	0.32 years	2.99 years
0.84	3.56 years	0.32 years	3.34 years

### PALYs for oxen: No controls

Sample data in Table 1 was used to estimate PALYs for the oxen population in this region beginning with levels 2, 3 and lastly 4.

Table 7 shows the numbers of YLD, YLL and PALYs lost for the 124 oxen when *D*_*w*_ = 0.33. At this level, the YLD is on average 0.27 years (approximately 3 months and a week). The YLL is approximately 2.61 years (approximately 31 months and 2 weeks). The number of PALYs lost for the 124 oxen in level 2 is 358.37 years. Hence, on average, a farmer loses 2.88 years (approximately 34 months and 3 weeks) of productivity per ox due to fatality of the disease.

**Table 7:** PALYs for oxen with *D*_*w*_ = 0.33.

Age group	*N*_*i*_	*a*_*i*_	*I*	*D*_*w*_	YLD	*N*_*d*_	*a*_*d*_	*L*	YLL	PALYs
0–4	6	2	1	0.33	4.09	6	3	11.45	71	75.09
5–9	19	7	1	0.33	11.77	19	8	8.36	138	149.77
10–15	25	12	1	0.33	10.87	25	13	5.59	87.17	98.04
16–20	31	18	1	0.33	4.75	31	19	3.13	19.95	24.70
20 +	43	20	1	0.33	2.47	43	21	2.35	8.30	10.77
Total	124				33.95	124			324.24	358.37

Table 8 shows the numbers of YLD, YLL and PALYs lost for the 124 oxen when *D*_*w*_ = 0.52. At this level, the YLD is on average 0.42 years (approximately 5 months). The YLL is approximately 2.61 years (approximately 31 months and 2 weeks). The number of PALYs lost for the 124 oxen in level 3 is 376.38 years. This implies that on average, a farmer loses 3 years (approximately 36 months and 2 weeks) of productivity per ox due to fatality of the disease.

**Table 8:** PALYs for oxen with *D*_*w*_ = 0.52.

Age group	*N*_*i*_	*a*_*i*_	*I*	*D*_*w*_	YLD	*N*_*d*_	*a*_*d*_	*L*	YLL	PALYs
0–4	6	2	1	0.52	6.08	6	3	11.45	71	77.08
5–9	19	7	1	0.52	17.48	19	8	8.36	138	155.48
10–15	25	12	1	0.52	16.15	25	13	5.59	87.17	103.32
16–20	31	18	1	0.52	7.05	31	19	3.13	19.95	27.00
20 +	43	23	1	0.52	5.20	43	21	2.35	8.30	13.50
Total	124				51.96	124			324.42	376.38

Table 9 shows the numbers of YLD, YLL and PALYs lost for the 124 oxen when *D*_*w*_ = 0.84. At this level, the YLD is on average 0.67 years (approximately 8 months). The YLL is approximately 2.61 years (approximately 31 months and 2 weeks). The number of PALYs lost for the 124 oxen in level 4 is 408.40 years. This implies that on average, a farmer loses 3.29 years (approximately 39 months and 2 weeks) of productivity per ox due to fatality of the disease.

**Table 9:** PALYs for oxen with *D*_*w*_ = 0.84.

Age group	*N*_*i*_	*a*_*i*_	*I*	*D*_*w*_	YLD	*N*_*d*_	*a*_*d*_	*L*	YLL	PALYs
0–4	6	2	1	0.84	9.83	6	3	11.45	71	80.83
5–9	19	7	1	0.84	28.24	19	8	8.36	138	166.24
10–15	25	12	1	0.84	26.10	25	13	5.59	87.17	113.27
16–20	31	18	1	0.84	11.40	31	19	3.13	19.95	31.35
20 +	43	20	1	0.84	8.41	43	21	2.35	8.30	16.71
Total	124				83.98	124			324.42	408.40

### PALYs for oxen: Tick control

The previous calculations of PALYs were done without considering intervention methods for tick control. We therefore sought to determine the PALYs gained when adopting tick control methods. Here we determined how many PALYs for 124 bulls will be averted when tick control is considered. Our calculation was similar to calculation of PALYS for cows with tick control.

Table 10 shows the PALYs lost from 124 oxen with tick control. The PALYs lost per ox with tick control is 0.29 years (approximately 3 months and 18 days). In Table 11, we present the PALYs with and without tick control for the individual cattle (ox). In the last column we highlight the PALYs averted as a result of tick control.

**Table 10:** PALYs for oxen with tick control.

Age group	*N*_*i*_	*a*_*i*_	*I*	*D*_*w*_	YLD	PALYs
0–4	6	2	12.02	0.10	6.20	6.20
5–9	19	7	8.98	0.10	14.95	14.95
10–15	25	12	6.23	0.10	11.48	11.48
16–20	31	18	3.40	0.10	2.81	2.81
20 +	43	20	2.61	0.10	1.65	1.65
Total	124					37.09

**Table 11:** PALYs averted per ox.

*D*_*w*_	PALYs (no treatment)	PALYs (treatment)	PALYs averted
0.35	2.80 years	0.29 years	2.51 years
0.52	3 years	0.29 years	2.69 years
0.84	3.29 years	0.29 years	years

### PALYs for bulls: No controls

We calculated the number of PALYs lost for 86 bulls. Our aim was to determine how many PALYs are averted when considering treatment.

Table 12 shows the calculation of PALYs lost for 86 bulls when considering disability weight from level 2 (*D*_*w*_ = 0.33). At this level, the YLL is on average 0.39 years (approximately 4 months and 3 weeks). The YLD is approximately 3.72 years (approximately 44 months and 2 weeks). The number of PALYs lost for 86 bulls is 354.08 years, therefore the average PALYs lost per bulls is 4.11 years implying that on average, a farmer loses 4.11 years (approximately 49 months and 1 weeks) of productivity per bull due to fatality of the disease.

**Table 12:** PALYs for bulls with *D*_*w*_ = 0.33.

Age group	*N*_*i*_	*a*_*i*_	I	*D*_*w*_	YLD	*N*_*d*_	*a*_*d*_	*L*	YLL	PALYs
0–4	4	2	1	0.33	4.09	4	3	10.53	69.48	73.57
5–9	13	7	1	0.33	11.77	13	8	7.79	134.85	146.62
10–15	17	12	1	0.33	10.87	17	13	5.72	87.85	98.72
16–20	22	18	1	0.33	4.75	22	19	3.00	19.64	24.39
20 +	30	20	1	0.33	2.47	30	21	2.30	8.31	10.78
Total	86				33.95	86			320.13	354.08

Table 13 shows the calculation of PALYs lost for 86 bulls when considering disability weight from level 3 (*D*_*w*_ = 0.52). At this level, the YLL is on average 0.60 years (approximately 7 months). The YLD is approximately 3.72 years (approximately 44 months and 2 weeks). The number of PALYs lost for 86 bulls is 372.09 years, therefore the average PALYs lost per bulls is 4.33 years implying that on average, a farmer loses 4.33 years (approximately 51 months and 2 weeks) of productivity per bull due to fatality of the disease.

**Table 13:** PALYs for bulls with *D*_*w*_ = 0.52.

Age group	*N*_*i*_	*a*_*i*_	*I*	*D*_*w*_	YLD	*N*_*d*_	*a*_*d*_	*L*	YLL	PALYs
0–4	4	2	1	0.52	6.08	4	3	10.53	69.48	75.56
5–9	13	7	1	0.52	17.48	13	8	7.79	134.85	152.33
10–15	17	12	1	0.52	16.15	17	13	5.72	87.85	104
16–20	22	18	1	0.52	7.05	22	19	3.00	19.64	26.69
20+	30	23	1	0.52	5.20	30	21	2.30	8.31	13.51
Total	86				51.96	86			320.13	372.09

Table 14 shows the calculation of PALYs lost for 86 bulls when considering disability weight from level 4 (*D*_*w*_ = 0.84). At this level, the YLL is on average 0.98 years (approximately 11 months and 2 weeks). The YLD is approximately 3.72 years (approximately 44 months and 2 weeks). The number of PALYs lost for 86 bulls is 372.09 years, therefore the average PALYs lost per bulls is 4.70 years (approximately 56 months) implying that on average, a farmer loses 4.70 years of productivity per bull due to fatality of the disease.

**Table 14:** PALYs for bulls with *D*_*w*_ = 0.84.

Age group	*N*_*i*_	*a*_*i*_	*I*	*D*_*w*_	YLD	*N*_*d*_	*a*_*d*_	*L*	YLL	PALYs
0–4	4	2	1	0.84	9.83	4	2	10.53	69.48	79.31
5–9	13	7	1	0.84	28.24	13	7	7.79	134.85	163.09
10–15	17	12	1	0.84	26.10	17	12	5.72	87.85	113.95
16–20	22	18	1	0.84	11.40	22	19	3.00	19.64	31.04
20+	31	23	1	0.84	8.41	41	31	2.30	8.31	16.72
Total	86				83.98	86			320.13	404.11

### PALYs for bulls: Tick control

The previous calculation of PALYs did not account for tick control interventions. In light of this, we investigated the potential PALYs that could be gained by implementing tick control measures. Specifically, we assessed the number of PALYs that could be averted through tick control in 86 bulls. Our calculation also was similar to calculation of PALYs for cows with tick control.

Table 15 shows us the PALYs lost from 86 bulls when considering treatment. The PALYs lost per bull during the treatment is 0.44 years (approximately 5 months and a week). Table 16 shown us the PALYs averted calculation per bull in various levels of disability. In Table 16, we present the PALYs with and without tick control for the individual cattle (bull). In the last column we highlight the PALYs averted as a result of tick control.

**Table 15:** PALYs for bulls with tick control.

Age group	*N*_*i*_	*a*_*i*_	*I*	*D*_*w*_	YLD	PALYs
0–4	4	2	11.09	0.10	7.19	7.19
5–9	13	7	8.23	0.10	14.90	14.90
10–15	17	12	6.10	0.10	11.59	11.59
16–20	22	18	3.30	0.10	2.60	2.60
20 +	30	20	2.83	0.10	1.71	1.71
Total	86					37.99

**Table 16:** PALYs averted per bull.

Disability Weight	PALYs (no treatment)	PALYs (treatment)	PALYs averted
0.35	4.11 years	0.44 years	3.67 years
0.52	4.33 years	0.44 years	3.89 years
0.84	4.69 years	0.44 years	4.25 years

## Discussion

From our analysis, the productivity years of a cow reared by resource-poor livestock owners lost due to the burden of ticks and TBDs in Eastern Cape province of South Africa falls in the approximate range 24%-28%. However, from this study, we have confirmed that introducing tick control reduces the loss to the range 2.5%-3.5% of their lifespan productivity, an indication that tick control will save around 21.5%-24.5% of years of productive life of cows. In case of oxen, approximately 18.13%-21.87% of productivity years of oxen are lost due to burden of ticks and TBDs. However, introducing tick control reduces the loss to approximately 2.15%-2.85% of their lifespan productivity. Thus, tick control will save around 15.98%-19.02% of years of productive life of oxen. In case of bulls, approximately 29.6%-34.4% of productivity years of bulls are lost due to burden of ticks and TBDs. However, introducing tick control reduces the loss to approximately 3.3%-4.7% of their lifespan productivity. Thus, tick control will save around 26.3%-29.7% of years of productive life of bulls. It is therefore clear that the use of ticks and TBD control early enough will improve cattle productivity and hence socio-economic welfare of resource-poor rural farming communities in the eastern Cape province of South Africa.

In calculating PALYs for burden of ticks and TBDs, our calculations required many estimates associated with the PALYs calculation (e.g., the age at onset *a*_*i*_, expected age of death with and without treatment *a*_*d*_ and the duration of the disability *I*) and assumptions (disability weight *D*_*w*_ with and without treatment, choice of age weight and at what rate, choice of discounting and at what rate *r*). All these decisions affect the difference in PALYs with and without treatment. Our application was also able to show the relative contribution of YLL and YLD to total PALYs. Presentation of the full calculation also allows others to insert alternative values to re-estimate PALYs. This would be particularly helpful if, for example, we wished to generalize the results to another setting where lifespan differed, or where disability resulting from the condition was considered to be better or worse.

The calculations have also shown that age at onset of disease is an important factor determining variation of number of PALYs averted. The pattern of variation is mostly dictated by the shape of the age weighting function. PALYs are decreased when disease starts in the early years of life (or in the older ages of life) and is of short duration. It is increased when the disease starts in later years of life up to old age. These conclusions are based on the use of the age weighting function originally proposed in this research. The results would have been different if we had made use of a different age weighting function. This calculation is helpful in cost-effective analysis [34], when researchers want to compare different strategies or programs for prevention and control of the same disease. The cost-effectiveness calculation is particularly useful when relating different programs that are focusing on the same disease or goal.

Adding the societal burden (non-monetary) measure of disease to the existing economic (monetary) measure has several advantages. Firstly, it provides a more comprehensive assessment of the actual cost of a disease to society. While the economic impact is significant, it only captures part of the full range of social, cultural, and environmental impacts a disease can have on communities. By considering the non-monetary aspects of disease burden, policymakers and decision-makers can better understand the overall impact of a disease on society. Secondly, considering societal burdens can help identify vulnerable populations disproportionately affected by a disease. For example, specific communities may have cultural or religious practices impacted by the loss of livestock or limited access to veterinary services in the event of a disease outbreak. By understanding the non-monetary impacts of disease, policymakers can develop targeted interventions to address these specific needs. Thirdly, incorporating non-monetary disease burden measures can help build public trust and support for disease control and prevention efforts. When stakeholders see that the full range of impacts is being considered in decision-making processes, they are more likely to feel that their concerns and needs are being considered. In conclusion, adding a societal burden measure to existing economic measures provides a complete understanding of the impact of a disease on society, helps to identify vulnerable populations, and builds public trust and support for disease control and prevention efforts.

## Conclusion

The findings generated from the PALYs approach developed in this study are helpful in projections for the future burden of any livestock disease and may be used as a basis in policy formulation and decision-making by various stakeholders, and hence a priority in animal health economics. We recommend that a classification of livestock diseases of national economic importance should consider both the societal burden (non-monetary) and economic impact instead of the common practice of only considering the economic (monetary) impact. Adding a societal burden measure to existing economic measures provides a holistic understanding of the impact of a disease on society especially in resource-limited settings where the livestock value goes beyond monetary value.

## Supporting information

S1 File Questionnaire document. (PDF)

S2 File The collected data file. (XLSX)

S3 File The formula for calculating the sample size of the livestock owners. (PDF)

S4 File This presents the basic procedure for calculating PALYs for cattle using a suitable example and provides an illustrative example. (PDF)

S1 Table Definition of disability weight (*D*_*w*_) for cattle amongst rural livestock owners in Eastern Cape Province, South Africa 2013. (PDF)

S2 Table Standard lifespan for cattle (cows). (PDF)

S3 Table Standard lifespan for cattle (oxen and bulls). (PDF)
