# Supplementary material for: Modelling the burden of disease for cattle–A case of ticks and tick-borne diseases in cattle in a rural set-up in South Africa
Source: PLoS One. 2023 Oct 20;18(10):e0293005. doi: 10.1371/journal.pone.0293005 (PMC10588883; doi:10.1371/journal.pone.0293005)
Supplement: S1 File — (PDF) [file pone.0293005.s001.pdf]

A.2 Questionnaire

Questionnaire number: .... Community name: ....

Enumerator name: .... Name of respondent:.....................

Municipality name: .... Date:...

1. How many animals do you have?

Class	Cows	Oxen	Bulls
Number			

2. Why do you keep animals?

Monetary/Commercial value	Rank (5-highest & 1-least)	Social value	Rank (5-highest & 1-least)
Meat/Growth		Social status	
Milk		Dowry	
Drought power		Cultural ceremonies	
sale		Other (specify)	

3. What characteristics do you use to assess the general condition of your animals?

Characteristic	Rank/Importance in classification (5-high & 1-least)

4. What is the general condition of your animals?

Excellent	Good	Poor	very poor

5. How long do your animals usually live?

Class	Cows	Oxen	Bulls
Age			

6. At what age do animals become productive for each of the following product series, indicate the age when your animals start and, stop being useful. Also indicate that is most useful?

Monetary/Commercial value	Start	Most useful	Stop
Meat/Growth			
Milk			
Drought power			
sale			

7. At what age do animals become productive for each of the following product series, indicate the age when your animals start and, stop being useful. Also indicate that is most useful?

Social value	Start	Most useful	Stop
Social status			
Dowry			
Cultural ceremonies			
Others (specify)			

8. What are the most common causes of cattle mortality? (Rank 1-5 with 1-most important cause of mortality and 5-least important).

Class	Cows	Oxen	Bulls
Diseases			
Old age			
Lack of forage/Drought			
Theft			
Others (specify)			

9. What are the most common diseases in your area? List.

Disease	Rank (5 1 with 5-most common & 1-least common)
Red water	
Heart water	
Mastitis	
Lumpy skin	
Anthrax	
Others(specify)	

10. Are ticks common in your area? If yes during which months do you see them a lot? ....

11. Do ticks have any effect on your animals? If yes, go to number 12. ....

12. From the list of uses of your animals, how would you rank the impact of ticks on the uses of your animals? Effect (1-insignificant, 2-mild, 3-severe).

Monetary/Commercial value	Effect	Social value	Effect
Meat/Growth		Social status	
Milk		Dowry	
Drought power		Cultural ceremonies	
sale		Other (specify)	

13. Do you control ticks and tick-borne diseases on your animals? If yes, go to number 14. ....

14. What control measures do you use? ....

15. In a period of one year, how many animals do you treat against ticks and tick-borne diseases? ....

16. If you dont treat your sick animals, how long will they survive?

....

17. What is the cost of the control measures you use against ticks, in a period of one year?

....

18. Do you seek specialist advice for tick control or when animals are suffering from diseases caused by ticks? If yes, who do you consult and how much do you pay for the service?

....

19. What is the total amount of money you use in tick control?

....

20. How many animals do you lose in total in a period of one (1) year? and

Class	Cows	Oxen	Bulls
Number			

21 At what age?

Class	Cows	Oxen	Bulls
Age			

22. How many animals do you lose in a year due to diseases caused by ticks? and

Class	Cows	Oxen	Bulls
Number			

23. At what age?

Class	Cows	Oxen	Bulls
Age			
