# Supplementary material for: Modelling the burden of disease for cattle–A case of ticks and tick-borne diseases in cattle in a rural set-up in South Africa
Source: PLoS One. 2023 Oct 20;18(10):e0293005. doi: 10.1371/journal.pone.0293005 (PMC10588883; doi:10.1371/journal.pone.0293005)
Supplement: S3 File — (PDF) [file pone.0293005.s003.pdf]

Sample Size Formula

The formula for calculating the sample size of the livestock owners based on the given assumptions is:

$$n = \frac{(Z^2 \times p \times q)}{E^2}, \tag{1}$$

where n is the required sample size, Z is the Z-score corresponding to the desired confidence level (for 95% confidence level, Z = 1.96), p is the estimated proportion of the population with the characteristic of interest (unknown, so assumed to be 0.5 for maximum sample size), $q = 1 - p$.
