# Supplementary material for: Modelling the burden of disease for cattle–A case of ticks and tick-borne diseases in cattle in a rural set-up in South Africa
Source: PLoS One. 2023 Oct 20;18(10):e0293005. doi: 10.1371/journal.pone.0293005 (PMC10588883; doi:10.1371/journal.pone.0293005)
Supplement: S4 File — (PDF) [file pone.0293005.s004.pdf]

Example

Assume that, at the age of 8, a cow gets sick with red-water disease which disables it for a certain period of time but after treatment it is in remission for 2 years. After 2 years it suffers from an onset of the same disease which disables it substantially and it dies as a result at age 10.

We wish to calculate the PALYs for this cow. In order to do this, we need to calculate the YLD and YLL.YLD: Assume that its disability is weighted as 0.4320 (disability weight for red-water). Since it lasts for 2 years (10 − 8 years)

$$\text{YLD for cattle} = 1 \times 2 \times 0.4320 = 0.86 \text{ years } (\approx 9 \text{ months}).$$

YLL: From Table 1, the cow that dies at age of 10 loses 8.22 years of expected life. Using equation (3), the PALYs for the cow in question becomes

$$\text{PALYs for cow} = 0.86 + 8.22 = 9.08 \text{ years.}$$

In this case, the number of PALYs lost due to the burden of disease is 9.08 years. This is the total productive time lost due to disability and premature death.

We revert to Example 1 this time taking discounting into account. First, we shall calculate YLD for the cow:

$$\text{YLD} = \frac{1 \times 0.4320 \times \left[1 - e^{-(0.13 \times 2)}\right]}{0.13} = 0.76 \text{ years}$$

In terms of YLL for the cow, we substitute standard lifespan for cow at age 10 from Table 1 ($L = 8.22$) to obtain

$$\text{YLD} = \frac{1 \times \left[1 - e^{-(0.13 \times 8.22)}\right]}{0.13} = 5.05 \text{ years}$$

Finally, the YLD and the YLL are summed up according to equation (3) to obtain

$$\text{PALYs} = 0.76 + 5.05 = 5.81 \text{ years.}$$

The burden of disease in this case in terms of PALYs is 5.81 years. Taking discounting into consideration has reduced the productive time lost due to disability and premature death from 9.08 years to only 5.81 years.

Now we return to Example 1 to calculate PALYs considering age weighting and discounting.

YLD: By substituting the values $a_i = 8, I = 2, r = 0.13, D_w = 0.432$ and $N_i = 1$

$$\begin{aligned} &\text{YLD for cow} \\ &= 0.20\left(-0.25\frac{\text{erf}\left(\frac{2 \times 0.01(8+2)+0.13}{2\sqrt{0.01}}\right) + \text{erf}\left(\frac{2 \times 0.01 \times 8 + 0.13}{2\sqrt{0.01}}\right)}{4\sqrt{0.01^3}}\right) \\ &+ 0.20\left(\frac{-e^{-(8+2)(0.01(8+2)+0.13)} + e^{-8(0.01 \times 8 + 0.13)}}{2 \times 0.01}\right) \\ &= 1.49 \text{ years.} \end{aligned}$$

Similarly, YLL is calculated by substituting $a_d = 10, L = 8{,}22, r = 0.13$ and $N_d = 1$ into equation (14) to yielded

YLD for cow

$$= 0.60\left(-0.33\frac{\operatorname{erf}\left(\frac{2\times 0.01(10+8.22)+0.13}{2\sqrt{0.01}}\right)+\operatorname{erf}\left(\frac{2\times 0.01\times 10+0.13}{2\sqrt{0.01}}\right)}{4\sqrt{0.01^3}}\right)$$

$$+ 0.60\left(\frac{-e^{-(10+8.22)(0.01(10+8.22)+0.13)}+e^{-10\times(0.01\times 10+0.13)}}{2\times 0.01}\right)$$

$= 6.51$ years.

By using equation (3), the number of PALYs for the cow is

$$\text{PALYs for the cow} = 1.49 + 6.51 = 8.00 \text{ years.}$$

Taking both age weighting and discounting into account has resulted into the productive time lost due to disability and premature death to now lie somewhere in between the previous two values as depicted in Table 4.

In summary, example 1 presented the basic procedure for calculating PALYs for cattle using a suitable example and provided an illustrative example demonstrating the process with and without considering discounting rate and age weighting. Table 4 summarizes the key findings of our analysis, presenting the PALY values obtained under different scenarios considered.

Table 4. Summary results of PALYs calculation with/without consideration of discounting and age weighting (Example 1)

Scenario	Age weighting	Discount rate	YLD	YLL	PALYs
PALYs [0; 0]	No	0	0.86	8.22	9.08
PALYs [0; 0.13]	No	0.13	0.76	5.05	5.81
PALYs [1; 0.13]	Yes	0.13	1.49	6.51	8.00
