# Supplementary material for: Modelling the burden of disease for cattle–A case of ticks and tick-borne diseases in cattle in a rural set-up in South Africa
Source: PLoS One. 2023 Oct 20;18(10):e0293005. doi: 10.1371/journal.pone.0293005 (PMC10588883; doi:10.1371/journal.pone.0293005)
Supplement: S1 Table — (PDF) [file pone.0293005.s005.pdf]

Disability Weight

Table 3: Definition of disability weight (D_w) for cattle amongst rural livestock owners in Eastern Cape Province, South Africa 2013

Levels	Description	D_w
1	Beef production [(500 - 600kg for oxen), (300 - 516kg for bulls), (320 - 440 kg for cows)].<br>Milk production [5 - 6 litres per day].<br>Draught power [3 - 5hrs for cows, 5 - 6hrs for oxen].<br>Social status [acceptable].<br>Dowry payment [acceptable].<br>Cultural ceremonies [acceptable].	0
2	Beef production [(400 - 499kg for oxen), (260 - 299kg for bulls), (280 - 319kg for cows)].<br>Milk production [3.5 - 4.9 litres per day].<br>Draught power [2 - 3hrs for cows, 3 - 4hrs for oxen].<br>Social status [not very acceptable for reason of loss of condition].<br>Dowry payment [not very acceptable for reason of loss of condition].<br>Cultural ceremonies [not very acceptable for reason of loss of condition].	$0.01 - 0.33$
3	Beef production [(300 - 399kg for oxen), (220 - 259kg for bulls), (200 - 239kg for cows)].<br>Milk production [2 - 3.4 litres per day].<br>Draught power [1 - 2hrs for cows, 2 - 3hrs for oxen].<br>Social status [not acceptable for reason of being diseased].<br>Dowry payment [not acceptable for reason of being diseased].<br>Cultural ceremonies [not very acceptable for reason of being diseased].	$0.34 - 0.66$
4	Beef Production [(200 - 299kg For Oxen), (180 - 219kg For Bulls), (240 - 279kg For Cows)].<br>Milk Production [1 - 1.9 litres per day].<br>Draught Power [0 - 1hrs For Cows, 1 - 2hrs For Oxen].<br>Social Status [not Acceptable For Reason Of Being Thin And Diseased].<br>Dowry Payment [not Acceptable For Reason Of Being Thin And Diseased].<br>Cultural Ceremonies [not Very Acceptable For Reason Of Being Thin And Dis- eased]	$0.67 - 0.99$
