# Supplementary material for: Modelling the burden of disease for cattle–A case of ticks and tick-borne diseases in cattle in a rural set-up in South Africa
Source: PLoS One. 2023 Oct 20;18(10):e0293005. doi: 10.1371/journal.pone.0293005 (PMC10588883; doi:10.1371/journal.pone.0293005)
Supplement: S2 Table — (PDF) [file pone.0293005.s006.pdf]

Lifespan

Table 1. Standard lifespan for cattle (Cows)

x	n_x	$d_x = n_x - n_{n+1}$	$l_x = \frac{n_x}{n_0}$	$q_x = \frac{d_x}{n_x}$	$L_x = \frac{(l_x + l_{x+1})}{2}$	$T_x = T_{x-1} - L_{x-1}$	$e_x = \frac{T_x}{l_x}$
0	116	5	1.00	0.04	0.98	12.83	12.83
1	111	5	0.96	0.05	0.94	11.85	12.38
2	106	5	0.91	0.05	0.89	10.91	11.94
3	101	4	0.87	0.04	0.85	10.02	11.51
4	97	5	0.84	0.05	0.81	9.17	10.96
5	92	5	0.79	0.05	0.77	8.35	10.53
6	87	5	0.75	0.06	0.73	7.58	10.11
7	82	5	0.71	0.06	0.69	6.85	9.70
8	77	5	0.66	0.06	0.64	6.17	9.29
9	72	4	0.62	0.06	0.60	5.53	8.90
10	68	4	0.59	0.06	0.57	4.92	8.22
11	64	4	0.55	0.06	0.53	4.35	7.89
12	60	5	0.52	0.08	0.50	3.82	7.38
13	55	2	0.47	0.04	0.47	3.32	7.01
14	53	5	0.46	0.09	0.44	2.86	6.25
15	48	4	0.41	0.08	0.40	2.42	5.85
16	44	4	0.38	0.09	0.36	2.03	5.34
17	40	3	0.34	0.08	0.33	1.66	4.83
18	37	3	0.32	0.08	0.31	1.33	4.18
19	34	4	0.29	0.12	0.28	1.03	3.50
20	30	5	0.26	0.17	0.24	0.75	2.90
21	25	5	0.22	0.20	0.19	0.51	2.38
22	20	3	0.17	0.15	0.16	0.32	1.85
23	17	7	0.15	0.41	0.12	0.16	1.09
24	10	10	0.09	1.00	0.04	0.04	0.50
25	0	0	0.00	0.00	0.00	0.00	0.00
$\sum$		116	13.33		12.83		
