# Supplementary material for: Modelling the burden of disease for cattle–A case of ticks and tick-borne diseases in cattle in a rural set-up in South Africa
Source: PLoS One. 2023 Oct 20;18(10):e0293005. doi: 10.1371/journal.pone.0293005 (PMC10588883; doi:10.1371/journal.pone.0293005)
Supplement: S3 Table — (PDF) [file pone.0293005.s007.pdf]

Lifespan

Table 2. Standard lifespan for cattle (Oxen and Bulls)

Age x	Standard lifespan (Oxen)	Standard lifespan (Bulls)
0	13.15	12.34
1	12.59	11.64
2	12.02	11.09
3	11.45	10.53
4	10.78	9.96
5	10.19	9.39
6	9.59	8.82
7	8.98	8.23
8	8.36	7.79
9	7.74	7.35
10	7.02	6.92
11	6.65	6.50
12	6.23	6.10
13	5.59	5.72
14	5.08	5.38
15	4.41	4.70
16	3.93	4.17
17	3.62	3.80
18	3.40	3.30
19	3.13	3.00
20	2.61	2.83
21	2.35	2.30
22	1.97	1.75
23	1.33	1.17
24	0.50	0.50
